# Supplementary material for: Programmable liquid-core fibers: Reconfigurable local dispersion control for computationally optimized ultrafast supercontinuum generation
Source: Nat Commun. 2025 Aug 25;16:7918. doi: 10.1038/s41467-025-63213-8 (PMC12379246; doi:10.1038/s41467-025-63213-8)
Supplement: Supplementary file 1 — Supplementary Information [file 41467_2025_63213_MOESM1_ESM.pdf]

Supplementary Information to:

Programmable Liquid-Core Fibers: Reconfigurable Local Dispersion Control for Computationally Optimized Ultrafast Supercontinuum Generation

Johannes Hofmann[1], Ramona Scheibinger[1], Bennet Fischer[1], Mario Chemnitz[1, 2], and Markus A. Schmidt[1, 3, *]

[1]Leibniz Institute of Photonic Technology, Albert-Einstein-Str. 9, 07745 Jena, Germany
[2]Friedrich Schiller University Jena, Institute of Applied Optics and Biophysics, Philosophenweg 7, 07743 Jena, Germany
[3]Friedrich Schiller University Jena, Otto Schott Institute of Materials Research, Fraunhoferstr. 6, 07743 Jena, Germany
[*]markus-alexander.schmidt@uni-jena.de

Supplementary Note 1: Validation of step-like temperature transition using thermal diffusion simulations

To simulate the temperature distribution inside the fiber, we performed finite element simulations on a 2D silica glass block using boundary conditions that closely resemble the experimental configuration. In the following, we first describe the simulation methodology used (Sec. A), then evaluate the steepness of the temperature gradient for the specific LCF configuration employed in this study (Sec. B), and finally assess how geometric and material parameters influence the resulting temperature distribution (Sec. C).

A: Description of simulations

To reveal the internal temperature distribution within the LCF sample, we conducted numerical simulations of the steady-state temperature profile in a 2D silica glass block using a Python-based finite-difference scheme. The model discretizes the Laplace equation on a rectangular grid, with periodic boundary conditions applied along the fiber axis (z-direction). To replicate the experimental setup, a pitch of $\Lambda = 5.6\,\mathrm{mm}$ was chosen, corresponding to twice the length of a single Peltier element (2.8 mm). The bottom boundary ($x = 0$) is maintained at a spatially periodic temperature profile ($T_0 = 20\,°\mathrm{C}$, $T_1 = 80\,°\mathrm{C}$) to simulate external heating, implementing Dirichlet boundary conditions. The top boundary ($x = 125\,\mu\mathrm{m}$, matching the experimental LCF diameter) employs a Robin (mixed) boundary condition to account for convective heat transfer to the surrounding air ($T_{\mathrm{air}} = 20\,°\mathrm{C}$). The numerical solution is obtained using a custom Python code by assembling and solving the resulting sparse linear system for the interior points, with all boundary conditions directly incorporated. This approach provides a physically realistic model of the thermal behavior of an infinitely extended silica glass block in contact with air on one side, closely mirroring experimental conditions and incorporating the thermal conductivity of silica ($k_{\mathrm{silica}} = 1.31\,\mathrm{W\,m^{-1}\,K^{-1}}$)[1] as well as the heat transfer coefficient at the silica–air interface ($h_{\mathrm{heat}} = 10\,\mathrm{W\,m^{-2}\,K^{-1}}$)[2].

B: Temperature distribution in the LCF used in this study

The resulting 2D temperature distribution in Fig. S1(a) demonstrates that the transverse temperature variation along the x-axis is relatively minor, primarily due to the small outer diameter of the fiber

($x = 125\,\mu$m). This effect becomes even more apparent when examining the longitudinal temperature profile at the midpoint of the block ($x = 62.5\,\mu$m, Fig. S1(b)), which shows only slight deviations from the ideal step-like profile at the curve edges.

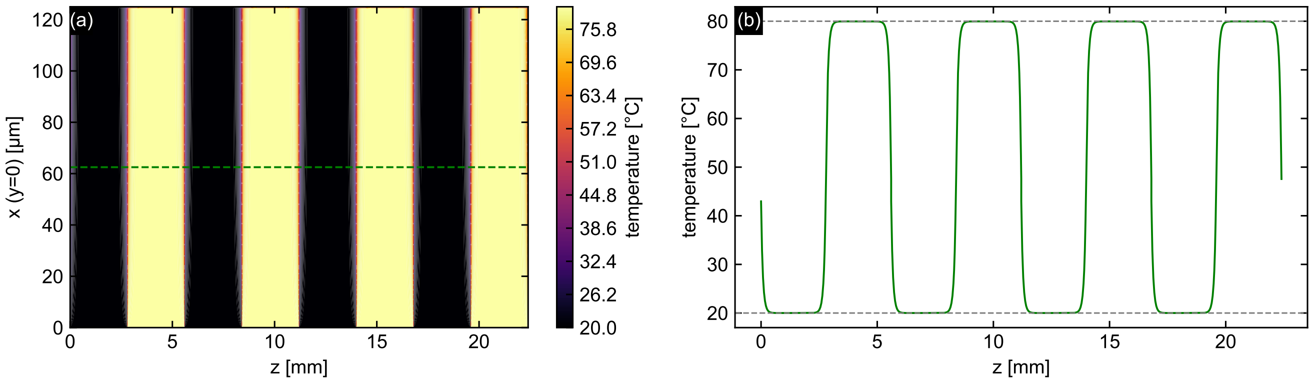

Figure S1. Simulated temperature distribution within a silica glass block, with periodic Dirichlet boundary conditions applied at the bottom ($x = 0$) and Robin boundary conditions at the top surface ($x = 125\,\mu$m). To reflect the experimental configuration, the periodicity Λ was set to 5.6 mm, matching twice the length of one Peltier element (2.8 mm). Further simulation details are described in the text. (a) Temperature map in the xz-plane at $y = 0$. (b) Longitudinal temperature profile at half the size of the glass block ($x = 62.5\,\mu$m, indicated by horizontal dashed green lines in (a)). The horizontal dashed gray lines indicate the maximum and minimum temperatures considered (20 °C and 80 °C).

C: Temperature distribution in different LCF configurations

Impact of materials: To assess the impact of material parameters on the temperature distribution, it is useful to refer to the simulations described in Sec. 1B, which are fundamentally based on solving the Laplace equation to determine the steady-state temperature profile. These simulations use periodic boundary conditions along the z-axis to represent periodic heating, with Dirichlet and Robin boundary conditions applied at the bottom and top interfaces to simulate heating from below and exposure to an open environment above. The only direct influence of material properties in these simulations arises in the Robin boundary condition at the top, which models convective heat dissipation into air using the thermal conductivity of silica and the heat transfer coefficient at the silica–air interface. As shown in Fig. S1(a), the temperature distribution along the x-direction remains largely uniform, with only minimal deviations near the uppermost region of the glass ($x \leq 125\,\mu$m), indicating that the impact of material properties is generally minor.

Impact of geometric features: Figure S2 presents simulated longitudinal temperature distributions at the center of the glass block along the x-direction, corresponding to the location of the fiber core, for different geometric configurations. As shown in Fig. S2(a), small block extensions (e.g., 80 µm, 125 µm) result in temperature profiles that closely approximate a step-like function, whereas larger extensions lead to smoother profiles due to increased thermal diffusion. Figure S2(b) shows temperature distributions for different periodicities (pitch values), indicating that for comparably large pitches - such as the experimental value of $\lambda = 5.6$ mm - the step-like character of the temperature distribution is preserved, and cross-talk between regions of different temperature remains minimal. Noticeable smoothing only appears

for much smaller pitch values. These findings confirm that, under the experimental parameters used (pitch $\Lambda = 5.6\,\mathrm{mm}$, extension $x = 125\,\mu\mathrm{m}$), the temperature distribution remains sharply step-like and cross-talk between heating elements is small, thereby validating the experimental design.

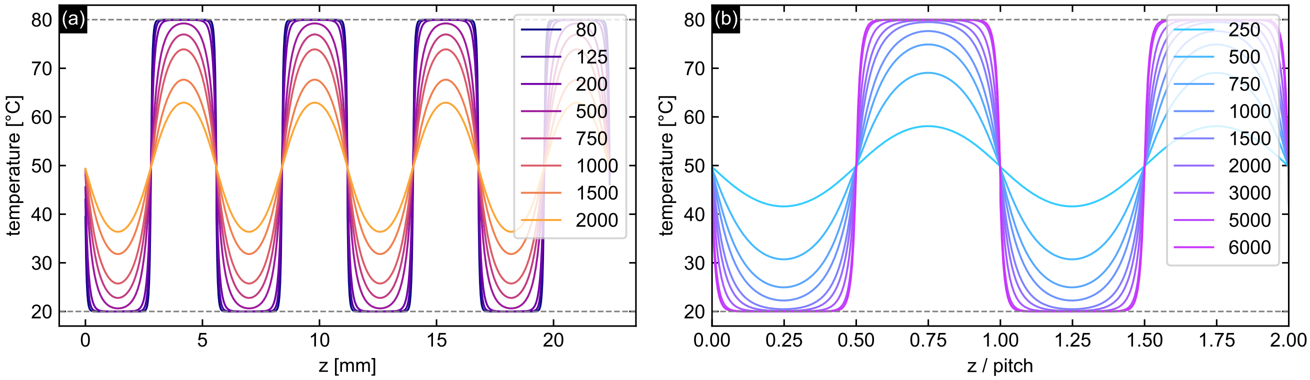

Figure S2. Simulated longitudinal temperature distributions at half the extension (along the x-axis) of the glass block, corresponding to the fiber core position (see Sec. 6A for simulation details). (a) Temperature profiles for different glass block extensions (indicated in the legend in micrometers, $\Lambda = 5.6\,\mathrm{mm}$). (b) Temperature profiles for different periodicities (pitch values shown in the legend in micrometers). The x-axis is normalized to the respective pitch to allow direct comparison of the profiles.

Supplementary Note 2: Progression of the best objective value during the optimization of the shown simulation examples

Figure S3 shows the search progression of the best found objective value during optimization, normalized to the maximum value, for the simulative optimizations shown in Fig. 4a and Fig. 5a in the main text. Both examples clearly demonstrate strong optimization (reduction of the objective value) during the optimization process.

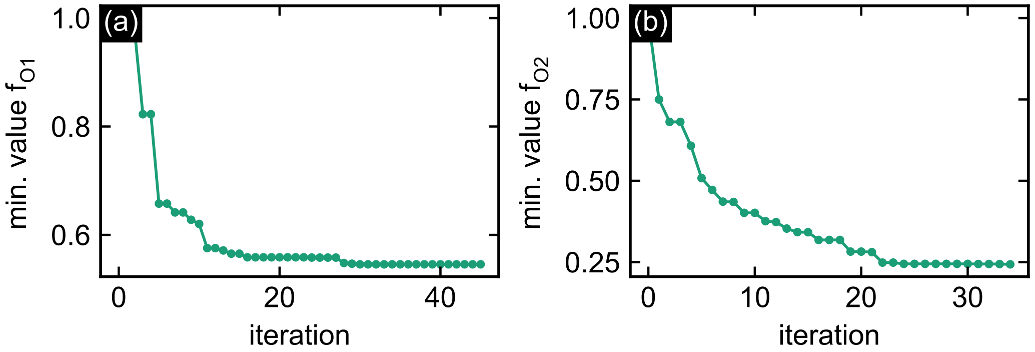

Figure S3. Progression of the best objective value during the optimization of the shown simulation examples in the main text.

Supplementary Note 3: Additional example of spectral maximizing in simulations

In the following, we present two additional examples of maximizing selected intervals. First, Fig. S4 shows another example of optimization with 2 target intervals, which exhibits a mean enhancement factor of 10 according to the definition in Eq. 3 in the main text. As before, the relevant region of the final spectrum, the pulse evolution along the fiber with corresponding temperature, and the progression of the best found objective value during optimization are shown. Additionally, Fig. S5 analogously presents an example of maximization with 3 target intervals. Following the same procedure, a mean enhancement factor of 4 is obtained.

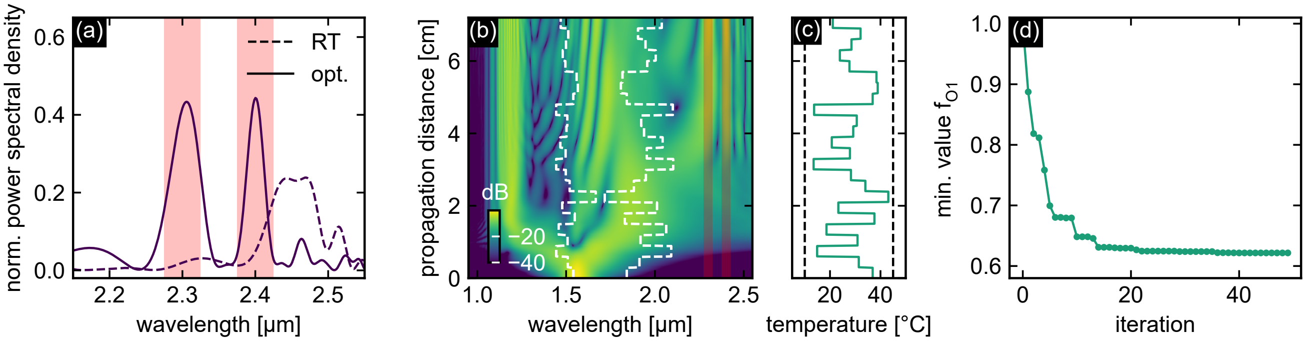

Figure S4. Simulation results of the PSO-based optimization to maximize the spectral power in two selected spectral intervals. (a) Example of an optimized output spectrum (solid line) compared to the unoptimized configuration (room temperature, dashed line). Interval width $\Delta\lambda = 50\,\text{nm}$, $\lambda_{t,1} = 2300\,\text{nm}$, $\lambda_{t,2} = 2400\,\text{nm}$). The light red areas indicate the optimization intervals. (b) Corresponding spatio-spectral pulse evolution using an optimized dispersion distribution. The curves are normalized by the maximum power of the input pulse. The white dashed lines indicate the ZDWs. (c) Corresponding temperature distributions. (d) Progression behavior of the best objective value during the optimization process showing a consecutive decrease, i.e. optimization of the process.

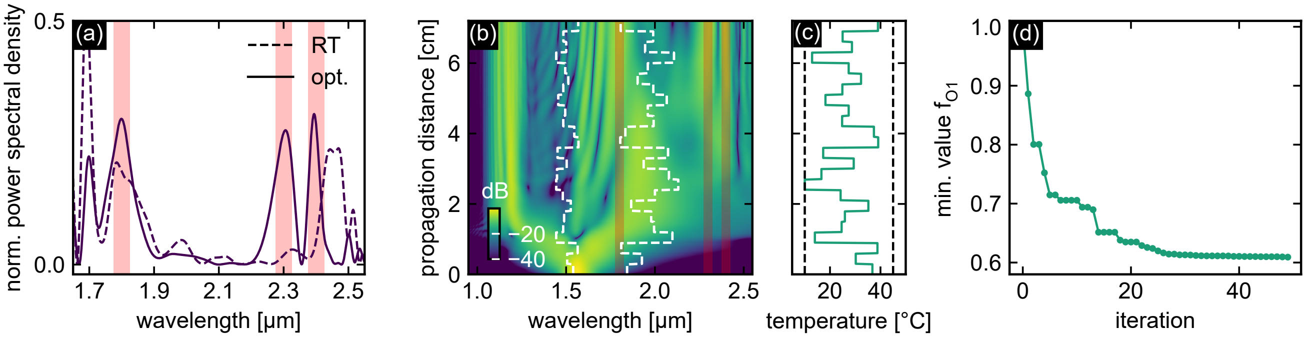

Figure S5. Simulation results of the PSO-based optimization to maximize the spectral power in three selected spectral intervals. (a) Example of an optimized output spectrum (solid line) compared to the unoptimized configuration (room temperature, dashed line). Interval width $\Delta\lambda = 50\,\text{nm}$, $\lambda_{t,1} = 1800\,\text{nm}$, $\lambda_{t,2} = 2300\,\text{nm}$, $\lambda_{t,3} = 2400\,\text{nm}$. The light red areas indicate the optimization intervals. (b) Corresponding spatio-spectral pulse evolution using an optimized dispersion distribution. The curves are normalized by the maximum power of the input pulse. The white dashed lines indicate the ZDWs. (c) Corresponding temperature distributions. (d) Progression behavior of the best objective value during the optimization process showing a consecutive decrease, i.e. optimization of the process.

Supplementary Note 4: Additional example of spectral flattening in simulations

In the following Fig. S6, we present another example of spectral flattening with a target wavelength $\lambda_t = 1400\,\text{nm}$ and interval width $\Delta\lambda = 200\,\text{nm}$. The calculated degree of flatness according to the definition Eq. 5 given in the main manuscript is 0.08 with a power enhancement of factor 5.

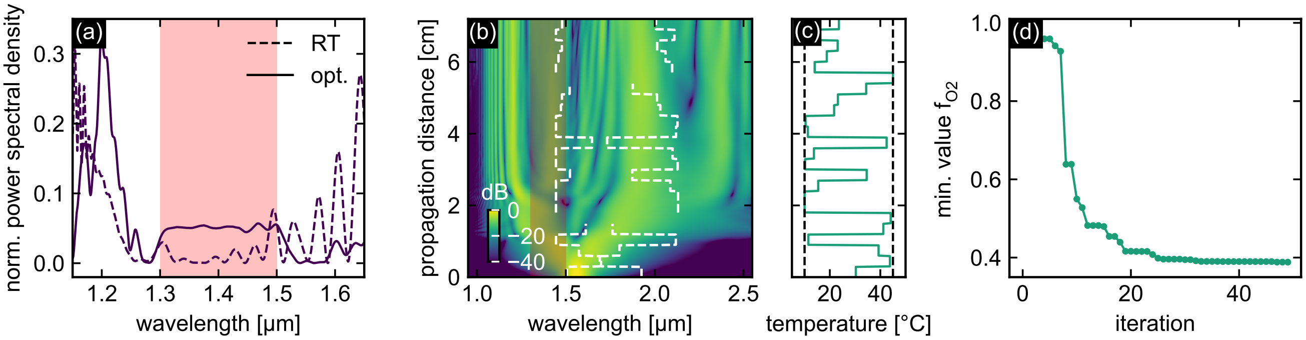

Figure S6. Simulation results of PSO-based optimization aiming at flat spectral output at high power levels. (a) Example of an optimized output spectrum (solid line) compared to the unoptimized configuration (room temperature, dashed line). Interval width $\Delta\lambda = 200\,\text{nm}$, $\lambda_t = 1400\,\text{nm}$. The light red area refers to the optimization interval. (b) Corresponding spatio-spectral pulse evolution with the white dashed lines marking the ZDWs. The curves are normalized by the maximum power of the input pulse. (c) Corresponding temperature distributions, where the vertical black dashed line indicates the minimal and maximal possible temperatures.

Supplementary Note 5: Progression of the best objective value and spectral stability during the optimization of the shown experimental examples

As mentioned in the main text, the stability of the optical excitation of the HOM and the associated stability of the generated output spectrum are essential for algorithm-controlled optimization. Therefore, we present the spectra measured during optimization at the end of each iteration at room temperature, i.e., with deactivated Peltier elements, corresponding to the experimental examples shown in the main text. Additionally, for each example, the progression of the best found objective value during optimization is shown. Figure S7 contains the examples of maximization with 2 target intervals, while Fig. S8 shows the flattening of a broad target interval. The spectra are shown from the start of the measurement until reaching the best solution. It is clearly visible that over the corresponding time period, the HOM was excited and spectral features remained acceptably constant. Minor changes, such as in Fig. S7(a,e), occurred within a

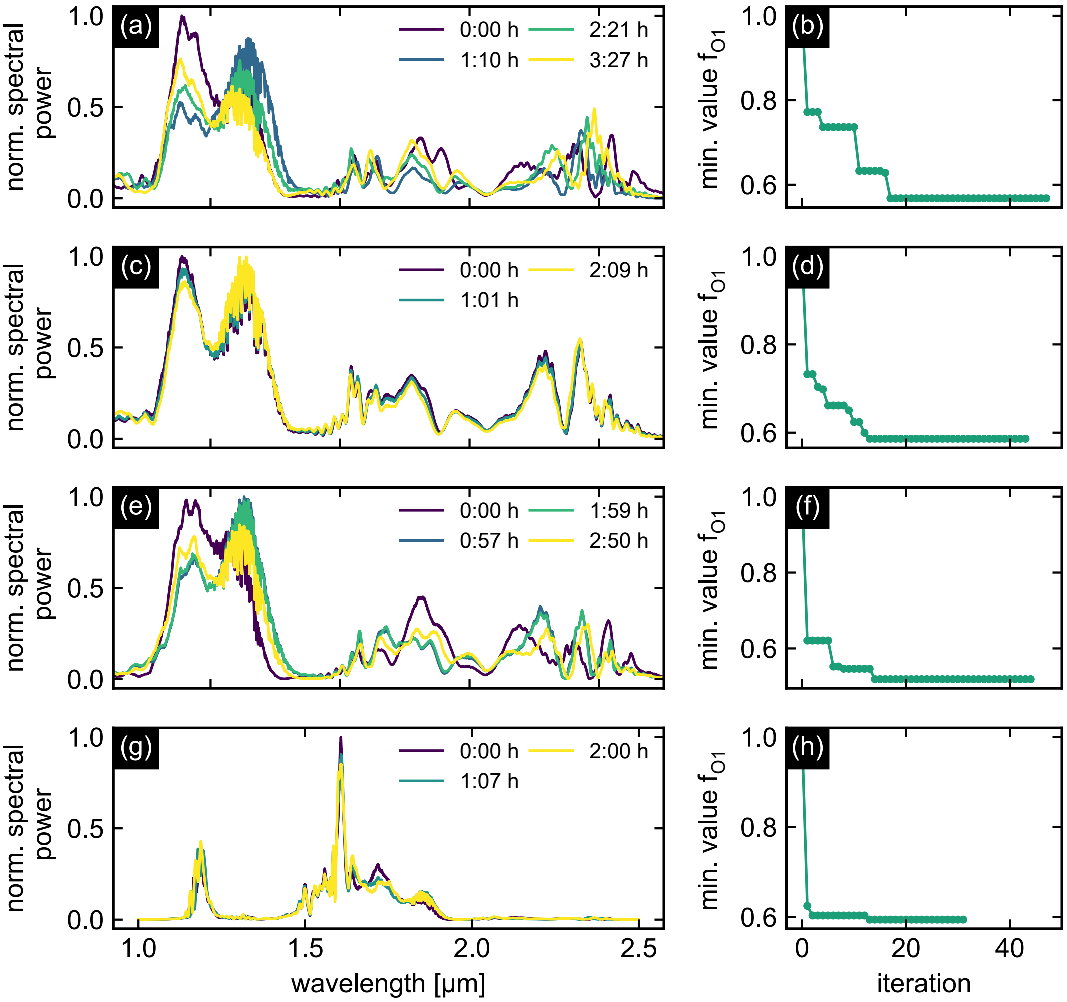

Figure S7. Spectral stability and progression of best objective value of the examples of spectral power maximization in two intervals shown in the main text. The left column (a,c,e,g) shows the time stability of the generated output spectra at room temperature. The right column (b,d,f,h) shows the progression of the best objective value of the optimization process.

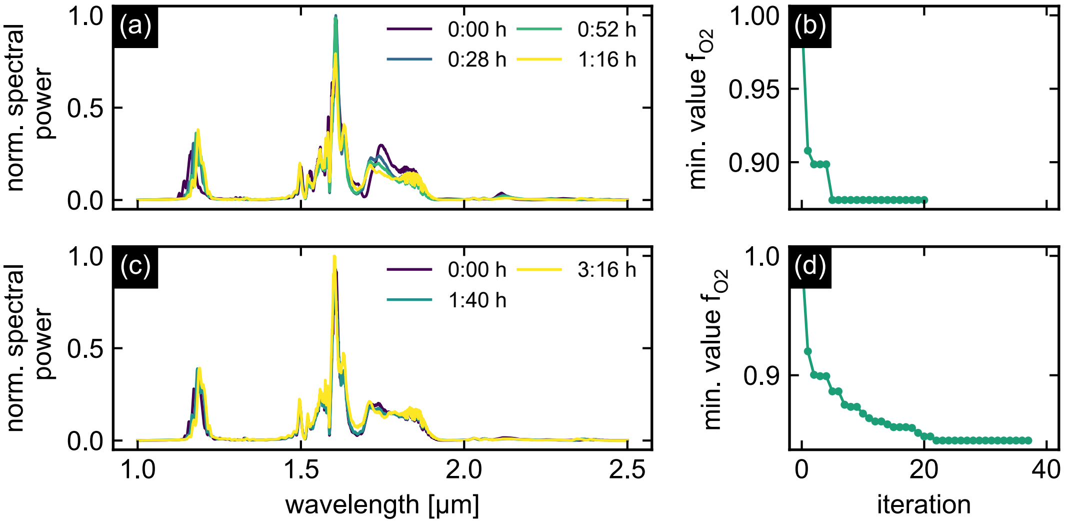

Figure S8. Spectral stability and progression of best objective value of the examples of spectral flattening shown in the main text. The left column (a,c) shows the time stability of the generated output spectra at room temperature. The right column (b,d) shows the progression of the best objective value of the optimization process.

small time window at the beginning of the measurement period, allowing subsequent optimization under constant conditions. The right columns show that an optimized solution was found step by step in a few iterations with the help of the algorithm.

Supplementary Note 6: Impact of realistic temperature variations on spectral output

To illustrate the impact of experimental deviations from idealized conditions, additional numerical simulations were performed that include realistic temperature variations along the fiber. These simulations are based on the same parameters used in the main manuscript (sech² pulse shape, 30 fs pulse duration, center wavelength 1560 nm, peak power 10 kW, fiber length 7.2 cm, core diameter 3.92 µm, excitation of the TM_{01} mode, and temperature profile as shown in Fig. 4c of the main text), but with introduced $\pm 2\,°C$ temperature variations in individual heating segments. As shown in Fig. S9, even small local temperature deviations lead to spectral differences in the resulting supercontinuum. These findings highlight the strong temperature sensitivity of the system and emphasize the challenge of achieving exact agreement between numerical and experimental results.

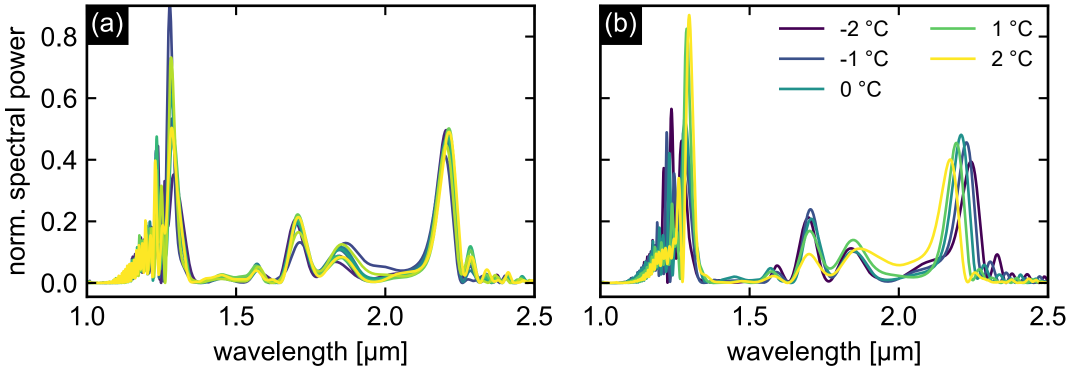

Figure S9. Nonlinear pulse propagation simulations showing generated output spectra for different configurations that include deviations from the ideal temperature distribution (see text for details). (a) Random temperature variations between $-2\,^{\circ}$C and $2\,^{\circ}$C added to each temperature interval. (b) A constant temperature offset (values indicated in the legend) added to each temperature interval.

Supplementary Note 7: Material and structural considerations for temperature programmable fibers

A critical parameter in the programmable fiber concept is the use of a material with a very high thermo-optic coefficient (TOC), enabling modal dispersion to be manipulated via tailored temperature patterns. To provide a comprehensive overview of suitable materials, Tab. S1 summarizes TOCs for a range of photonic materials relevant to fiber optics, including liquids, chalcogenide glasses, and semiconductors. Notably, liquid CS_2 exhibits a very large negative TOC ($-8\cdot10^{-4}\,K^{-1}$)[3], making it extremely responsive to temperature changes, while other liquids such as benzene[4] and C_2Cl_4[3] also show strong negative TOC values.
For comparison, silicon has a positive TOC of $1.9\cdot10^{-4}\,K^{-1}$ [5], which is significant for solid-state materials and similar in order of magnitude to CS_2. Germanium offers an even higher TOC than Si ($5\cdot10^{-4}\,K^{-1}$) [5,6], while its high absorption at near-IR wavelengths excludes it from waveguiding applications in this range. The chalcogenide glass $Ge_{33}As_{12}Se_{55}$ shows a positive TOC of $7.6\cdot10^{-5}\,K^{-1}$ [7,8], making it useful for mid-IR applications, though with lower temperature sensitivity than CS_2. Note that As_2S_3, already used in hybrid chalcogenide-silica fibers[9], has a much lower TOC[10], making it less suitable for temperature-sensitive applications. This comparison clearly justifies the choice of CS_2 as the core material, as it offers the highest TOC among feasible high-quality step-index fiber materials. Preliminary simulations investigating the temperature dependence of the group velocity dispersion (GVD) in liquid, semiconductor, and chalcogenide glass cylindrical core fibers with silica cladding reveal the highest temperature sensitivity for CS_2/silica fibers in higher-order modes - a finding that is currently under further investigation. Other fiber types exhibit substantially lower temperature dependence due to the complex interplay of refractive index, TOC, and waveguiding properties. At present, we consider the CS_2–silica step-index fiber to provide the highest temperature susceptibility, although ongoing research is aimed at more fully characterizing the temperature-dependent properties of alternative core materials. We are currently conducting additional simulation-based studies to address this question in detail.
We would also like to point out that LCFs can be realized with microstructured claddings to tailor the GVD profile. This was recently demonstrated by supercontinuum generation in a liquid-core microstructured optical fiber using CS_2 as the core material[11], which enabled a dispersion landscape with a zero-dispersion

wavelength for the fundamental mode near the telecom range - unlike conventional capillary-type fibers, which have less favorable dispersion properties for soliton fission. Further research is needed to determine whether tailored external temperature patterns can be effectively transferred to the fiber core in the presence of a holey cladding, and whether the temperature sensitivity of the guided modes - ideally the fundamental mode - can be further enhanced by the use of a microstructured cladding.

Table S1. Comparison of TOCs for various photonic materials relevant to fiber optics, including liquids, chalcogenide glasses, and semiconductors. The table summarizes the magnitude and sign of the TOCs, the measurement wavelengths, and the relevant literature sources.

Material	Class	dn/dT (K^{-1})	Transparency range
CS_2	liquid	$\sim -8 \cdot 10^{-4}$ [3]	VIS to NIR[11]
C_2Cl_4	liquid	$\sim -6 \cdot 10^{-4}$ [3]	VIS to NIR[11]
CCl_4	liquid	$\sim -3.9 \cdot 10^{-4}$ [3]	VIS to NIR[11]
Toluene	liquid	$\sim -5.5 \cdot 10^{-4}$ [4]	Up to 1.6 µm[12–15]
As_2S_3	chalcogenide glass	$\sim 9 \cdot 10^{-6}$ [10]	1 – 7 µm[16]
As_2Se_3	chalcogenide glass	$\sim 3.2 \cdot 10^{-5}$ [7]	2 – 13 µm[17]
$Ge_{33}As_{12}Se_{55}$	chalcogenide glass	$\sim 7.6 \cdot 10^{-5}$ [7,8]	1 – 15 µm[18]
Si	semiconductor	$\sim 1.9 \cdot 10^{-4}$ [5,6]	1 – 8 µm[17,19–22]
Ge	semiconductor	$\sim 5 \cdot 10^{-4}$ [5]	1.5 – 15 µm[17–19,21]
GaAs	semiconductor	$\sim 2.3 \cdot 10^{-4}$ [23–26]	1 – 16 µm[17,24]

Supplementary Note 8: Optical setup for temperature-controlled supercontinuum generation

Schematic representation of the experimental configuration as described in the Methods section of the main text. It includes an ultrafast laser source (1560 nm, 30 fs, 80 MHz), excitation optics for mode conversion into the TM_{01} mode, the pLCF with integrated heating elements, diagnostics, and a computer-controlled feedback loop. The coupling was actively stabilized using a piezo-controlled stage, enabling long-term stable TM_{01} excitation with approx. 20 % efficiency at 100 mW average power.

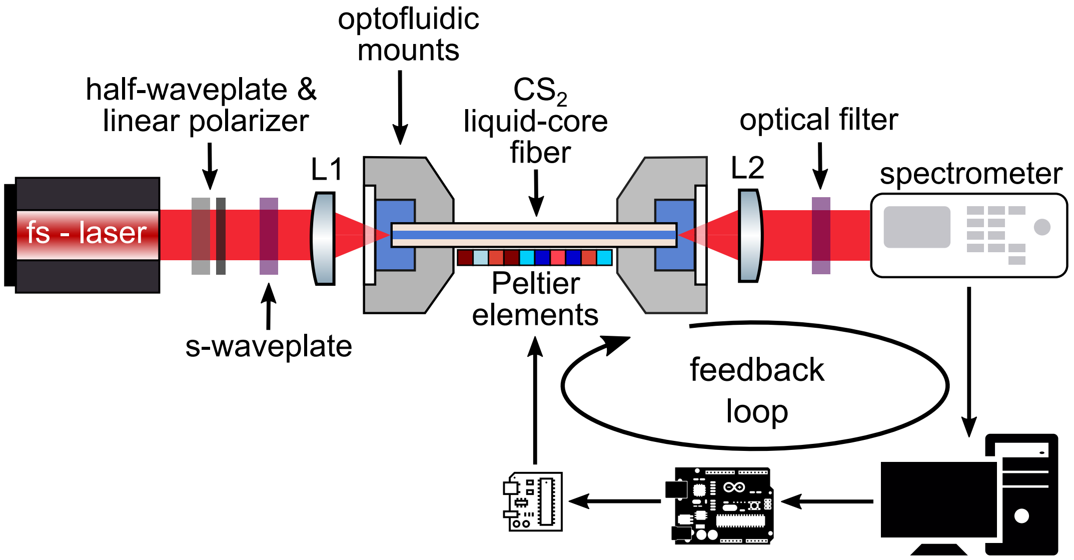

Figure S10. Schematic of the experimental setup used for computational optimized supercontinuum generation, including excitation optics, pLCF sample, temperature control, diagnostics, and computer-controlled optimization feedback loop. Note that the s-waveplate is used to create the polarization pattern of the TM_{01}-mode. The different colors of the Peltier elements below the pLCF indicate different temperatures.

Supplementary Note 9: Hyperparameters of particle swarm algorithm

Implementation and parameter settings of the particle swarm optimization as described in the Methods section of the main text.

Table S2. Settings of the PSO used for the optimization of the simulated and experimental spectra.

parameter	value
parameters	24
bounds (sim)	10 to 45 (temperature)
bounds (exp)	-1 to 1 (PWM)
swarm size	100 (sim), 50 (exp)
cognitive factor	1.3
social factor	1.8
inertia	0.5

References

1. Zhu, W., Zheng, G., Cao, S. & He, H. Thermal conductivity of amorphous SiO2 thin film: A molecular dynamics study. *Sci. Rep.* **8**, 10537 (2018), DOI: 10.1038/s41598-018-28925-6.
2. Dvurechensky, A. V., Petrov, V. A. & Yu Reznik, V. Spectral emissivity and absorption coefficient of silica glass at extremely high temperatures in the semitransparent region. *Infrared Phys.* **19**, 465–469 (1979), DOI: 10.1016/0020-0891(79)90060-5.

3. Pumpe, S., Chemnitz, M., Kobelke, J. & Schmidt, M. A. Monolithic optofluidic mode coupler for broadband thermo- and piezo-optical characterization of liquids. *Opt. Express* **25**, 22932–22946 (2017), DOI: 10.1364/OE.25.022932.

4. Samoc, A. Dispersion of refractive properties of solvents: Chloroform, toluene, benzene, and carbon disulfide in ultraviolet, visible, and near-infrared. *J. Appl. Phys.* **94**, 6167–6174 (2003), DOI: 10.1063/1.1615294.

5. Frey, B. J., Leviton, D. B. & Madison, T. J. Temperature-dependent refractive index of silicon and germanium. 62732J. DOI: 10.1117/12.672850.

6. Komma, J., Schwarz, C., Hofmann, G., Heinert, D. & Nawrodt, R. Thermo-optic coefficient of silicon at 1550 nm and cryogenic temperatures. *Appl. Phys. Lett.* **101**, 041905 (2012), DOI: 10.1063/1.4738989.

7. Gleason, B., Richardson, K., Sisken, L. & Smith, C. Refractive Index and Thermo–Optic Coefficients of Ge–As–Se Chalcogenide Glasses. *Int. J. Appl. Glass Sci.* **7**, 374–383 (2016), DOI: 10.1111/ijag.12190.

8. Nofziger, M. J. & Wolfe, W. L. Refractive Index Measurements Of AMTIR-1 At Cryogenic Temperatures. SPIE Proceedings, 118, DOI: 10.1117/12.964636 (SPIE, 1984).

9. Granzow, N. *et al.* Mid-infrared supercontinuum generation in As2S3-silica "nano-spike" step-index waveguide. *Opt. Express* **21**, 10969–10977 (2013), DOI: 10.1364/OE.21.010969.

10. Andrianov, A. V., Marisova, M. P. & Anashkina, E. A. Thermo-Optical Sensitivity of Whispering Gallery Modes in As2S3 Chalcogenide Glass Microresonators. *Sensors* **22** (2022), DOI: 10.3390/s22124636.

11. Junaid, S. *et al.* Supercontinuum generation in a carbon disulfide core microstructured optical fiber. *Opt. Express* **29**, 19891–19902 (2021), DOI: 10.1364/OE.426313.

12. Bertie, J. E., Jones, R. N., Apelblat, Y. & Keefe, C. D. Infrared Intensities of Liquids XIII: Accurate Optical Constants and Molar Absorption Coefficients between 6500 and 435 cm^{-1} of Toluene at 25°C, from Spectra Recorded in Several Laboratories. *Appl. Spectrosc.* **48**, 127–143 (1994), DOI: 10.1366/0003702944027516.

13. Myers, T. L. *et al.* Accurate Measurement of the Optical Constants n and k for a Series of 57 Inorganic and Organic Liquids for Optical Modeling and Detection. *Appl. Spectrosc.* **72**, 535–550 (2018), DOI: 10.1177/0003702817742848.

14. Chemnitz, M., Junaid, S. & Schmidt, M. A. Liquid–Core Optical Fibers—A Dynamic Platform for Nonlinear Photonics. *Laser Photonics Rev.* **17** (2023), DOI: 10.1002/lpor.202300126.

15. Plidschun, M., Chemnitz, M. & Schmidt, M. A. Low-loss deuterated organic solvents for visible and near-infrared photonics. *Opt. Mater. Express* **7**, 1122 (2017), DOI: 10.1364/OME.7.001122.

16. Théberge, F. *et al.* Mid-infrared nonlinear absorption in As2S3 chalcogenide glass. *Opt. Express* **24**, 24600–24610 (2016), DOI: 10.1364/OE.24.024600.

17. Lin, H. *et al.* Mid-infrared integrated photonics on silicon: a perspective. *Nanophotonics* **7**, 393–420 (2017), DOI: 10.1515/nanoph-2017-0085.

18. Hilton, A. Infrared Transmitting Glasses As Optical Materials In Passive Systems. 73–76. DOI: 10.1117/12.956043.

19. Soref, R. Mid-infrared photonics in silicon and germanium. *Nat. Photon* **4**, 495–497 (2010), DOI: 10.1038/nphoton.2010.171.

20. Boyd, I. W., Binnie, T. D., Wilson, J. I. B. & Colles, M. J. Absorption of infrared radiation in silicon. *J. Appl. Phys.* **55**, 3061–3063 (1984), DOI: 10.1063/1.333300.

21. Lord, R. C. Far Infrared Transmission of Silicon and Germanium. *Phys. Rev.* **85**, 140–141 (1952), DOI: 10.1103/PhysRev.85.140.2.

22. Becker, M. & Fan, H. Y. Optical Properties of Semiconductors. III. Infra-Red Transmission of Silicon. *Phys. Rev.* **76**, 1531–1532 (1949), DOI: 10.1103/PhysRev.76.1531.

23. Skauli, T. *et al.* Thermo-optic characterization of GaAs for quasi-phase-matched nonlinear-optical applications. 668. DOI: 10.1109/CLEO.2002.1034460.

24. Mourgelas, V., Sessions, N. P., Wilkinson, J. S. & Murugan, G. S. Modelling of a miniature mid-IR thermo-optic spectrometer on chip based on a $GaAs/In_{0.49}Ga_{0.51}P$ waveguide platform. *Opt. Commun.* **495**, 127044 (2021), DOI: 10.1016/j.optcom.2021.127044.

25. Della Corte, F. G., Cocorullo, G., Iodice, M. & Rendina, I. Temperature dependence of the thermo-optic coefficient of InP, GaAs, and SiC from room temperature to 600K at the wavelength of 1.5µm. *Appl. Phys. Lett.* **77**, 1614–1616 (2000), DOI: 10.1063/1.1308529.

26. Skauli, T. *et al.* Improved dispersion relations for GaAs and applications to nonlinear optics. *J. Appl. Phys.* **94**, 6447–6455 (2003), DOI: 10.1063/1.1621740.
